# A Simple Sample Preparation with HPLC-UV Method for Estimation of Clomipramine from Plasma

**Published:** 2010

**Authors:** Sayed Abolfazl Mostafavi, Reza Tahvilian, Masoumeh Dehghani Poudeh, Zeinab Rafeepour

**Affiliations:** a *Faculty of Pharmacy and Pharmaceutical Sciences and Isfahan Pharmaceutical Research Center, Isfahan University of Medical Sciences, Isfahan, Iran.*; b * School of Pharmacy, Kermanshah University of Medical Sciences, Kermanshah, Iran.*

**Keywords:** Clomipramine, Bioequivalency, Anafranil^®^, HPLC method

## Abstract

Clomipramine is a tricyclic antidepressant. Different methods for determination of clomipramine hydrochloride in plasma have been described. Most of these procedures favor the use of acidic back-extraction in extraction procedure and HPLC as the analytical technique. In this study, the clomipramine extraction procedure was modified and a direct injection to the column was performed to shorten the time of sample preparation considerably. Furthermore, the method was applied in bioequivalence study of new formulations of clomipramine in comparison with reference tablets.

The drug and internal standard were extracted from plasma with heptan : isoamyl alcohol (95:5) and re-extracted with 200 μL of orthophosphoric acid (0.3% v/v). The organic layer was discharged and analysis was performed on C_8_ reverse phase ODS2 HPLC column with a mobile phase, acetonitrile : water (75:25) and UV detection set at 215 nm. Additionally, a single dose study was carried out with a two-sequence, crossover block-randomized design for bioequivalence study. Clomipramine tablets (3 × 25 mg) of either formulations (reference or test products) were administered separately in two occasions to 12 fasting healthy male volunteers. Blood samples were taken prior to and at 9 points within 48 h after dose administration.

The retention time of internal standard (cisapride), clomipramine, and desmethyl clomipramine were 5.6 ± 0.2, 10.3 ± 0.3, and 9.5 ± 0.3 min, respectively. The standard curve covering the concentration ranges of 2.5-120 ng/mL was linear (r^2^ = 0.9950 and 0.9979) for clomipramine and desmethyl clomipramine. The co-efficient of variation for intra-day and inter-day accuracy and precision was less than 18.3%. The pharmacokinetic parameters C_max_ and T_max_ were obtained directly from plasma clomipramine concentrations. K_el _was estimated by log-linear regression and AUC was calculated by the linear trapezoidal rule. The pharmacokinetic parameters AUC and C_max_ were tested for equivalence after log-transformation of data. The 90% standard confidence intervals of the mean values for the test/reference ratios, AUC, and C_max_ were within the acceptable bioequivalence limits of 0.80-1.20.

These results indicated that the analytical method was linear and accurate. Test and reference formulations were found to be bioequivalent and therefore interchangeable.

## Introduction

**Figure 1:**
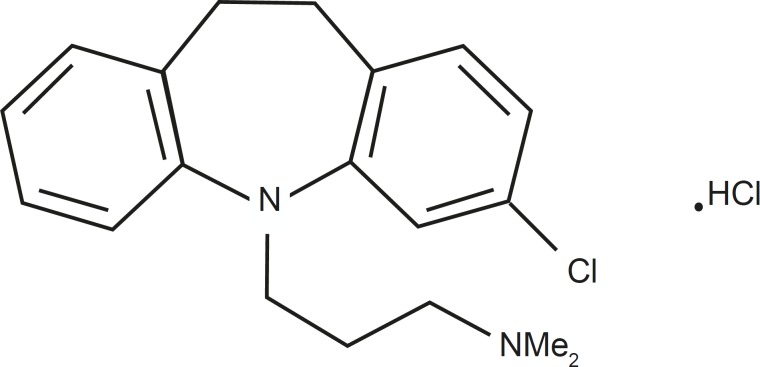
Chemical structure of clomipramine

Clomipramine (3-chloro-5-(3-dimethylamino propyl)-10,11-dihydro-5*H*-dibenz[b,f]azepine hydrochloride) is a dibenzazepine tricyclic antidepressant with actions and uses similar to those of amitriptyline (Figure 1).

 It has antimuscarinic properties and is also a potent serotonin reuptake inhibitor (1). Clomipramine (CMI) is one of the most sedating tricyclic (2- 4). The drug is recommended in obsessive compulsive disorders (OCD), in cataplexy associated with narcolepsy, and in depression when sedation is required (5-7). CMI is rapidly absorbed from the gastrointestinal tract and extensively desmethylated by first-pass metabolism in the liver to its primary active metabolite, desmethyl clomipramine (DMCMI) (8, 9). The metabolic pathways of both CMI and DMCMI include hydroxylation and N-oxidation. CMI is excreted in the urine, mainly in the form of its metabolites, either free or conjugated form (10-12). 

Most studies performed on determination of clomipramine, have measured only CMI. A few of them, however, have simultaneously determined its desmethyl and hydroxy-metabolites. Furthermore, these methods have accomplished a complex, expensive, and time-consuming three-step liquid–liquid extraction which requires more sophisticated equipment (13). These methods are time-consuming; therefore, they are cumbersome for bioavailability and bioequivalence studies. 

The pharmacokinetics of clomipramine has been documented elsewhere (14, 15), however, bioavailability issues have been an increasing concern to drug regulatory authorities once assessing the safety and efficacy of drug products. The increasing number of synonym drug products requires special attention terms of bioavailability issues. Hence, drug regulatory authorities have issued guidelines to ensure adequate bioavailability studies in new drug applications for synonym drugs (16). In this study, a simple and sensitive HPLC method using cisapride as internal standard (IS) with capability of simultaneously determining CMI and its N-desmethylated metabolites in human plasma were evaluated. Furthermore, the possible bioequivalency of a generic tablet of clomipramine (25 mg) made by an Iranian company in comparison to reference formulation was evaluated. 

## Experimental

*Reagents and solutions*

Clomipramine and desmethyl clomipramine powder were obtained from Farmaceutici (Italy) and cisapride from Jenson Co. (Belgium). Acetonitrile (HPLC grade), *n*-heptane (analytical grade, distilled before use), isoamyl-alcohol, sulfuric acid (all from Merck, Germany) were obtained from the local market. All other reagents and solutions were either HPLC or analytical grade. 

Clomipramine HCL tablets were obtained from a local pharmaceutical company. Anafranil^®^ (25 mg) tablets were from Novartis. 

*Chromatographic conditions*

A reversed phase HPLC method was developed to quantitate plasma levels of clomipramine. The apparatus used was a Jasco HPLC system (Japan), consisting of a model 980-PU intelligent solvent delivery pump, 7125-Rheodyne injector, a computerized system controller (with the Borwin software), and a UV- 975 detector set at 215 nm. Chromatographic separation was performed using a Perkin Elmer C-8 reverse phase ODS2 HPLC column. The mobile phase consisted of 75% acetonitrile, 25% water, and 0.01% triethylamine. The apparent pH of the mixed solvent system was adjusted to 4 **± **0.1 with a dilute solution of orthophosphoric acid. The aqueous phase was eluted at a flow rate of 1 mL/min, and effluent was monitored at 215 nm, at attenuation of 0.0005 and gain ×10. Quantitation was achieved by measurement of the peak area ratios of the drug to the internal standard.

*Sample preparation*

To 1 mL plasma in a 10 mL test tube, 0.5 mL NaOH (1 M) and 100 μL of IS (1 μg/mL) was added and extracted twice with 3 mL of extracted solvent [heptane: isoamylalcohol (95:5)]. Vortexed for 1 min and centrifuged at 2000 g for 5 min. Upper layer was separated in a tube and back extracted with 200 μL of 0.3% orthophosphoric acid. The organic layer was aspirated and 100 μL of the residue was injected to the column.

*Calibration procedure*

In order to make CMI standard concentrations, to 1 mL of blank plasma, 100 μL of CMI standard solution of CMT and DMCMI at concentrations of 2.5, 5, 10, 20, 40, 80 and 120 ng/100 μL plus 100 μL of IS 1μg /mL was added. All calibration samples were taken through the extraction procedure. The calibration curve was plotted using peak ratios of CMI/IS versus CMI concentrations. Final sample concentrations were calculated by determining the peak area ratio of CMI related to IS and comparing the ratio with the standard curve obtained after analysis of calibration samples.

*Extraction efficiency*

Recoveries of CMI from spiked samples were determined by comparing the peak areas obtained by extraction of freshly prepared plasma at concentrations of 2.5-120 ng/mL with those found by direct injection of an aqueous standard solution at equivalent concentrations.

*Precision*

*Within-day variability*

The within–day variability of the assay was determined by repeated analysis of quality control samples at concentrations ranging from 2.5 to 120 ng/mL on the same day.

*Between–day variability*

The between-day variability of the assay was determined by repeated analysis of quality control samples at concentrations ranging from 2.5 to 120 ng/mL on 3 different days.

*In-vivo study design*

Two separate groups consisted of twelve healthy non-smoking male volunteers weighing from 60-85 kg completed the studies (aged 21 to 27). The study was performed on the basis of medical history, clinical examinations, and laboratory tests including hematology, blood and biochemistry, and urine analysis. No subject had a history or evidence of hepatic, renal, gastrointestinal or hematological deviations or any acute or chronic disease or drug allergy. All volunteers were instructed to abstain from taking any medication and xanthin containing foods for at least 2 weeks prior to and during the study. No milk or dairy products were served during the study. Informed consent was obtained from the subjects after explaining the nature and purpose of the study. The ethics committee of the Iranian ministry of health approved the study and was conducted in accordance with good clinical practice guidelines. 

The protocols used were the conventional, two-sequence, crossover block-randomized design for bioequivalence study with six subjects in each of the treatment group. After an overnight fasting, all subjects received a single dose of three units of 25 mg tablets (reference or test product) randomly, with 250 mL of water. Food and drinks were not allowed until 2 h after ingestion of tablets. The same lunch and dinner was served at 5 and 12 h after dosing for all volunteers. 

*Clinical protocol*

Approximately 10 mL of blood samples were drawn into heparinized tube through an indwelling cannula before (0 h) and at 1, 2, 4, 5, 6, 8, 12, 24 and 48 h after dosing. The blood samples were centrifuged at 2000 rpm for 15 min and plasma was separated and kept frozen at -20˚C in coded glass tubes. 

After a period of 14 days the study was repeated in the same manner to complete the crossover design.

*Pharmacokinetic analysis*

Estimation and calculation of pharmacokinetic parameters were performed using MS Excel software. The maximum CMI concentration (C_max_) and the corresponding peak time (T_max_) were determined by inspection of the individual drug plasma concentration-time profiles. The elimination rate constant (K_el_) was obtained from the least square fitted terminal log-linear portion of the plasma concentration-time profile. The elimination half-life (T_1/2_) was calculated as 0.693/K_el_. The area under the curve to the last measurable concentration (AUC_0-t_) was calculated by the linear trapezoidal rule. The area under the curve extrapolated to infinity (AUC_0-∞_) was calculated by equation AUC_0-t_ + C_t_ / K_el_ where C_t_ is the last measurable concentration. 

*Statistical analysis*

For the purpose of bioequivalence analysis, AUC_0-t_, AUC_0-∞_, and C_max_ were considered as primary variables. The two-way ANOVA for crossover design was used to asses the effect of formulations, periods, sequences, and subjects on these parameters. A difference between two related parameters was considered statistically significant for a P-value equal to or less than 0.05. The 90% confidence interval of the ratio of pharmacokinetic parameters of logarithmically transformed were also estimated. All statistical analysis was performed using SPSS version 12. 

## Results

**Figure 2:**
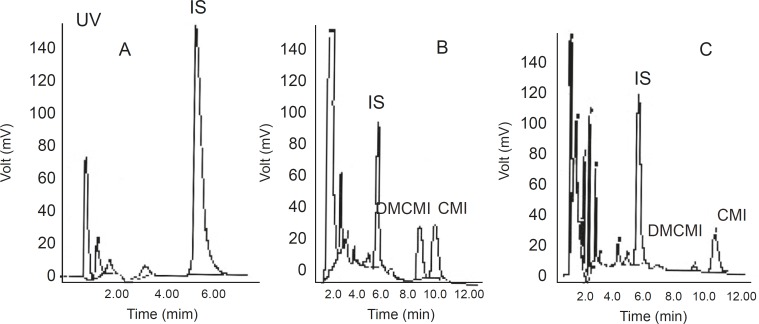
Chromatograms of blank human plasma and the internal standard (A), control plasma spiked with clomipramine and the internal standard (B), and plasma from a healthy subject 6 hours after ingestion of a clomipramine tablet (C).

No interfering peaks near the retention times of CMI, DMCMI, and IS were present in the chromatograms of blank or basal (time 0) plasma samples. Representative chromatograms are shown in Figure 2.

*Linearity*

**Figure 3:**
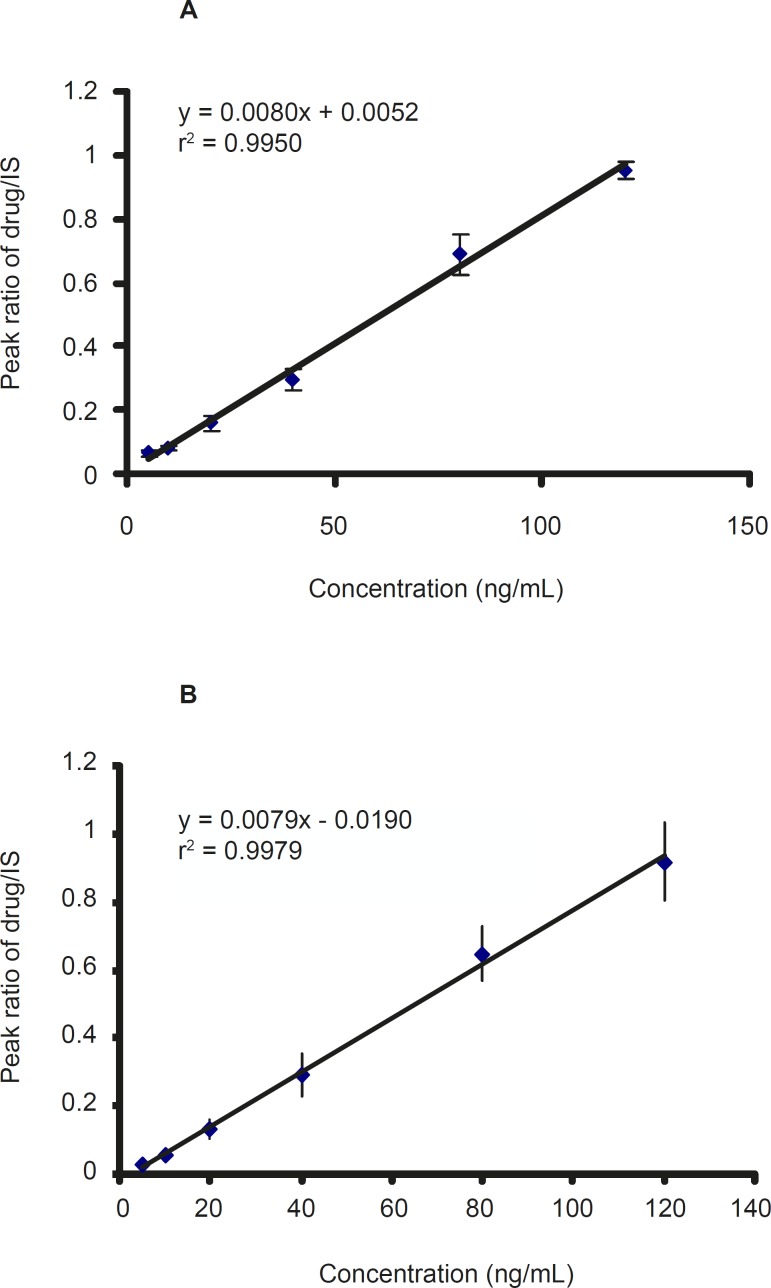
The calibration curve of A: clomipramine and B: desmethyl clomipramine in plasma

Calibration standard containing 2.5-120 ng/ mL was prepared from working solutions of CMI, DCMI, and blank plasma. The calibration curve was constructed by plotting the peak area ratio of CMI and DCMI to IS against the CMI and DCMI concentration in plasma. The ratios of the areas of the peaks of each compound versus IS were linearly related to the concentrations in the range encountered in drug monitoring. The calibration curves of CMI and DCMI after six-time replication are shown in Figure 3 which showed a good linearity within the examined concentration range. 

*Accuracy and precision*

The intra-day coefficients of variation (CV %) of CMI and its metabolite (DCMI) were between 3.09 and 18.33% and the inter-day coefficients of variation were between 7.25 and 14.51% for all compounds (Table 1 and 2). These results, therefore, validate the calibration curves used for each set of samples. 

**Table 1:** The intra–day variability of clomipramine and its major metabolite (desmethyl clomipramine) in plasma (n = 6).

**Clomipramine**				**Desmethyl clomipramine**			
**Added concentrations (ng/mL)**	**Experimental concentrations (ng/mL)**	**SD**	**CV (%)**	**Added concentrations (ng/mL)**	**Experimental concentrations (ng/mL)**	**SD**	**CV (%)**
5	6.45	1.12	17	5	5.80	0.20	4
10	10.24	1.13	11	10	10.32	1.10	11
20	19.96	2.45	12	20	18.21	1.59	9
40	35.28	3.46	10	40	38.28	4.33	11
80	80.43	8.33	10	80	77.63	11	15
120	115.48	3.58	3	120	98.82	17	18

SD: Standard deviation; CV: Coefficient of variation

**Table 2:** The inter–day variability of clomipramine and its major metabolite (desmethyl clomipramine) in plasma (n = 9).

**Clomipramine**				**Desmethyl clomipramine**			
**Added concentrations (ng/mL)**	**Experimental concentrations (ng/mL)**	**SD**	**CV (%)**	**Added concentrations (ng/mL)**	**Experimental concentrations (ng/mL)**	**SD**	**CV (%)**
5	5.52	0.63	12	5	5.52	0.48	9
10	10.29	1.49	15	10	10.04	1.39	14
20	19.68	2.66	14	20	18.74	1.58	8
40	39.92	3.87	10	40	40.34	5.03	12
80	87.29	7.30	8	80	83.37	9.85	12
120	118.63	8.60	7	120	121.30	12.9	11

SD: Standard deviation; CV: Coefficient of variation

*Sensitivity and recovery*

The limit of quantitation (sensitivity) and detection limit of the assay for CMI and DCMI was found to be less than 5 and 1 ng/mL, respectively.

The absolute recovery of CMI and its metabolite, which was determined by comparing the areas of the peaks of not-extracted standards with those of extracted standards according to the procedure described above, over the 5-80˚ ng/mL plasma concentration ranges, was between 62-72%.

*Pharmacokinetic parameters*

**Figure 4:**
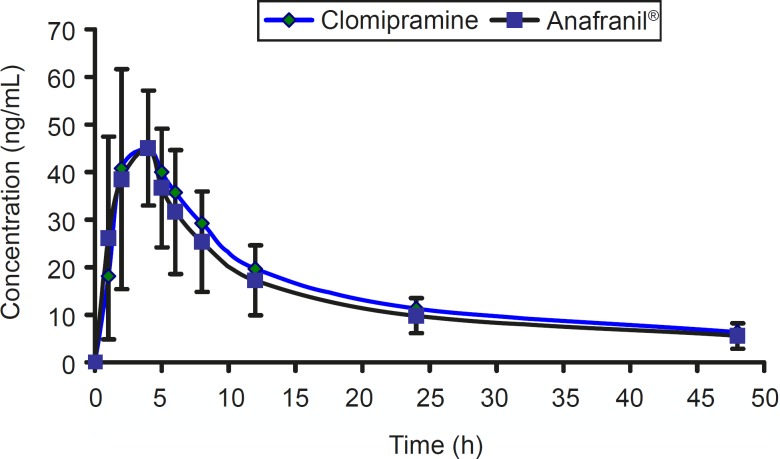
The mean concentration-time profiles of 3 × 25 mg clomipramine and Anafranil^® ^tablets.

The mean concentration-time profile for two brands of clomipramine is shown in Figure 4. 

The pharmacokinetic parameters of clomipramine 25 mg in comparison to the reference product is shown in Table 3. 

**Table 3:** Pharmacokinetic parameters of clomipramine hydrochloride after administration of 3 × 25 mg tablets

**Pharmacokinetic parameters**	**Clomipramine **	**Anafranil** ^®^	**Relative bioavailability (%)**
C_max_ (ng/mL)	46.42 ± 17.45	47.57 ± 18.79	1.00 ± 0.30
T_max_ (h)	3.75 ± 1.21	3.91 ± 1.08	NA
K_el_ (h ^-1^)	0.040 ± 0.014	0.044 ± 0.021	NA
T_1/2_ (h)	19.04 ± 6.59	18.08 ± 6.06	NA
AUC_0-48_ (ng.h/mL)	678.79 ± 171.43	618.17 ± 196.31	1.10 ± 0.30
AUC_0-∞_ (ng.h/mL)	836.31 ± 267.56	754.61 ± 283.01	1.10 ± 0.30

NA: Not applicable

For bioequivalence evaluation, various statistical modules were applied to AUC_0-t_, AUC_0-∞_, and C_max_ in accordance with current US Food and Drug Administration (FDA) guidelines (16). The 90% confidence intervals for AUC_0-t_, AUC_0-∞_, and C_max_ were within the range of 0.8-1.2 required by authorities. According to the mean plasma levels of the 12 subjects completing the study, the relative bioavailability of clomipramine tablet is shown in Table 3. The mean and standard deviation of both parameters for the two brands were found to be very close, indicating that the plasma profiles generated by clomipramine 3×25 mg is comparable to the reference standard (Anafranil^®^). Analysis of variance (ANOVA) for these parameters, after log-transformation of the data, showed no statistically significant differences (P > 0.05) between the two brands. In addition, 90% confidence interval demonstrated that the ratio of the AUC_0-t_ or AUC_0-∞_ of the two brands lie within the FDA accepted range 80-125%. For bioequivalence evaluation, t-student statistical procedure was performed on C_max_ values. This analysis showed that the two formulations of clomipramine 25 are not statistically different (P > 0.05) from each other. Moreover, 90% confidence interval also demonstrated that the ratio of the C_max_ lie within the FDA accepted range of 80-125%. 

## Discussion

Different methods for the determination of clomipramine hydrochloride in plasma have been described. Most of these procedures favor the use of acidic back-extraction in extraction procedure and HPLC as the analytical technique (13, 17, 18). In our experience, the acidic back-extraction and drying under nitrogen has been avoided and a modification was performed in extraction procedure. Extraction with 200 μL of 0.3% solution of ortho-phosphoric acid and direct injection to the column shortened the time of sample preparation significantly. 

Regulatory authorities usually require that one single dosing form to be administered. Exception is allowed if the plasma/serum levels are extremely low for the assay of the analytes. In those situations, a multiple of the dosage units are allowed to be administered for analytical reason. As reported by Pirola et al, CMI concentration is very low in plasma (19). Therefore, three units of CMI 25 mg dose were administered. During the experiment, the performance of measuring the concentration of the drug by HPLC could be increased. The blood was taken from volunteers by giving three units of 25 mg CMI from volunteers we used them for this protocol. 

All calculated pharmacokinetic parameter values were in good agreement with the previously reported values (9-11, 14, 15). Clomipramine was well tolerated by the volunteers in both phases of the study. All volunteers who started the study continued to the end and were discharged in good health. Unexpected incidents that could have influenced the outcome of the study did not occur. All formulations were readily absorbed from the gastrointestinal tract and clomipramine was measurable at the first sampling time (1 h) in almost all volunteers. 

The method was validated (20) using a linearity range of 2.5-120 ng/mL with a limit of detection less than 1 ng/mL and recovery ranged between 61.53-71.73%. The mean intra-day and inter-day CV were 10.68% (3.09-18.33%) and 10.82% (7.25-14.51%). 

The most important objective of bioequivalence testing is to assure the safety and efficacy of generic formulations. When two formulations of the same drug are equivalent in the rate and extent to which the active drug becomes available to the site of drug action, the two formulations are bioequivalent and thus considered therapeutically equivalent (21). In order to demonstrate bioequivalence, certain limits should be set depending on the nature of drug, patient population, and clinical end points. It is generally accepted that for basic pharmacokinetic characteristics, such as AUC and C_max_, the standard equivalence range is 0.8-1.25 (16). Pharmacokinetic and statistical results founded in our study showed Anafranil^®^ (3 × 25 mg) and clomipramine (3 × 25 mg) tablets are bioequivalent.

In conclusion, the HPLC method with modification in extraction procedure described above for the quantification of clomipramine was fast, sensitive, reliable, and reduced the time of clomipramine determination in plasma comparing to other methods. The statistical comparison of AUC_0-t_, AUC_0-∞_, and C_max_, obviously indicated no significant difference between the two brands of clomipramine tablets. In addition, 90% confidence intervals for the mean ratios (T/R) of AUC_0-t_, AUC_0-∞_, and C_max_ indicated that the reported values were entirely within the bioequivalence acceptance range of 80-125% (using log-transformed data). Based on the above mentioned pharmacokinetic and statistical results of this study, we can conclude that Anafranil^®^ (3 × 25 mg) and clomipramine (3 × 25 mg) tablets are bioequivalent. Thus, these products can be considered interchangeable in medical practice. 
